# Supplementary material for: Dose Optimization of Colistin: A Systematic Review
Source: Antibiotics (Basel). 2021 Nov 26;10(12):1454. doi: 10.3390/antibiotics10121454 (PMC8698549; doi:10.3390/antibiotics10121454)
Supplement: Supplementary file 1 [file antibiotics-10-01454-s001.zip › antibiotics-1452082-supplementary.pdf]

Table S1. Data extraction form for systematic review.

Review title or ID	
Study ID *(surname of first author and year first full report of study was published* e.g., *Smith 2001)*	

General information

Date form completed *(dd/mm/yyyy)*	
Name/ID of person extracting data	
Reference details	
Study author contact details	
Publication type *(e.g., full report, abstract, letter)*	
Notes:	

Study eligibility

Study Characteristics	Eligibility criteria *(Insert inclusion criteria for each characteristic as defined in the Protocol)*	Eligibility criteria met? Yes	No	Unclear	Location in text or source *(pg & ¶/fig/table/other)*
Type of study	Randomised Controlled Trial	☐	☐	☐	
	Case Reports	☐	☐	☐	
	Cohort Studies	☐	☐	☐	
Participants		☐	☐	☐	
Types of intervention		☐	☐	☐	
Types of comparison		☐	☐	☐	
Types of outcome measures		☐	☐	☐	
INCLUDE ☐		EXCLUDE ☐			
Reason for exclusion					
Notes:					

DO NOT PROCEED IF STUDY EXCLUDED FROM REVIEW

Characteristics of included studies

Methods

	**Descriptions as stated in report/paper**	**Location in text or source** *(pg no/fig/table/other)*

**Author and Year**		
**Study Design** *(e.g., parallel, crossover, non-RCT)*		
**Sample Size**		
**Characteristics of patients**		
**Dosing practice**		
**Clinical outcomes**		
**Dosing recommendations**		
**Ethical approval needed/obtained for study**	☐ Yes ☐ No ☐ Unclear	
**Notes:**		

Other information

Study funding sources *(including role of funders)*		
Possible conflicts of interest *(for study authors)*		
Notes:		

Quality Assessment Tool for included studies

Table S2. Newcastle-Ottawa Quality Assessment Form for Cohort Studies

Items	Number of stars
**Selection**	
Representative of exposed studies[A]	
Selection of non-exposed[B]	
Ascertainment of exposure[C]	
Demonstration of outcome[D]	
**Comparability**	
Comparability of cohort studies on basis of design[E]	
**Outcomes**	
Assessment of outcomes[F]	
Adequacy of follow-up[G]	
**Quality Score**	
A: *=truly representative or somewhat representative of average in target population<br>B: *=Drawn from the same community<br>C: *=Secured record or structured review<br>D: *=Yes, - = No<br>E: *= Study controls for age, gender, and other factors.<br>F: *=Record linkage or blind assessment, **=Both<br>G: *=follow-up of all subjects	

Risk of Bias assessment

**Domain**	**Risk of bias**			**Location in text or source** ***(pg no./fig/table/other)***
	**Low**	**High**	**Unclear**	
Random sequence generation *(selection bias)*	☐	☐	☐	
Allocation concealment *(selection bias)*	☐	☐	☐	
Blinding of participants and personnel *(performance bias)*	☐	☐	☐	
(if separate judgement by outcome(s) required)	☐	☐	☐	
Blinding of outcome assessment *(detection bias)*	☐	☐	☐	
(if separate judgement by outcome(s) required)	☐	☐	☐	
Incomplete outcome data *(attrition bias)*	☐	☐	☐	
(if separate judgement by outcome(s) required)	☐	☐	☐	
Selective outcome reporting? *(reporting bias)*	☐	☐	☐	
Other bias	☐	☐	☐	
Notes:				

Table S3: The Joanna Briggs Institute (JBI) Critical Appraisal Checklist for Case Reports

Major Components	Response Options			
	Yes	No	Unclear	Not Applicable
1. Were patient's demographic characteristics clearly described?	☐	☐	☐	☐
2. Was the patient's history clearly described and presented as a timeline?	☐	☐	☐	☐
3. Was the current clinical condition of the patient on presentation clearly described?	☐	☐	☐	☐
4. Were diagnostic tests or assessment methods and the results clearly described?	☐	☐	☐	☐
5. Was the intervention(s) or treatment procedure(s) clearly described?	☐	☐	☐	☐
6. Was the post-intervention clinical condition clearly described?	☐	☐	☐	☐
7. Were adverse events (harms) or unanticipated events identified and described?	☐	☐	☐	☐
8. Does the case report provide takeaway lessons?	☐	☐	☐	☐
Overall appraisal: Include □ Exclude □ Seek further info □				
